# Field Evaluation and Impact on Clinical Management of a Rapid Diagnostic Kit That Detects Dengue NS1, IgM and IgG

**DOI:** 10.1371/journal.pntd.0001993

**Published:** 2012-12-27

**Authors:** Anne-Claire Andries, Veasna Duong, Chantha Ngan, Sivuth Ong, Rekol Huy, Kim Kim Sroin, Vantha Te, Bunthin Y, Patrich Lorn Try, Philippe Buchy

**Affiliations:** 1 Institut Pasteur du Cambodge, Réseau international des Instituts Pasteur, Phnom Penh, Cambodia; 2 National Dengue Control Program, National Center of Parasitology, Entomology and Malaria Control, Ministry of Health, Phnom Penh, Cambodia; 3 Kampong Cham Provincial Hospital, Pediatric Department, Kampong Cham, Cambodia; 4 Takeo Provincial Hospital, Pediatric Department, Takeo, Cambodia; Oxford University Clinical Research Unit, Viet Nam

## Abstract

**Background:**

Dengue diagnosis is complex and until recently only specialized laboratories were able to definitively confirm dengue infection. Rapid tests are now available commercially making biological diagnosis possible in the field. The aim of this study was to evaluate a combined dengue rapid test for the detection of NS1 and IgM/IgG antibodies. The evaluation was made prospectively in the field conditions and included the study of the impact of its use as a point-of-care test for case management as well as retrospectively against a panel of well-characterized samples in a reference laboratory.

**Methodology/Principal Findings:**

During the prospective study, 157 patients hospitalized for a suspicion of dengue were enrolled. In the hospital laboratories, the overall sensitivity, specificity, PPV and NPV of the NS1/IgM/IgG combination tests were 85.7%, 83.9%, 95.6% and 59.1% respectively, whereas they were 94,4%, 90.0%, 97.5% and 77.1% respectively in the national reference laboratory at Institut Pasteur in Cambodia. These results demonstrate that optimal performances require adequate training and quality assurance. The retrospective study showed that the sensitivity of the combined kit did not vary significantly between the serotypes and was not affected by the immune status or by the interval of time between onset of fever and sample collection. The analysis of the medical records indicates that the physicians did not take into consideration the results obtained with the rapid test including for care management and use of antibiotic therapy.

**Conclusions:**

In the context of our prospective field study, we demonstrated that if the SD Bioline Dengue Duo kit is correctly used, a positive result highly suggests a dengue case but a negative result doesn't rule out a dengue infection. Nevertheless, Cambodian pediatricians in their daily practice relied on their clinical diagnosis and thus the false negative results obtained did not directly impact on the clinical management.

## Author Summary

Dengue is a potentially life-threatening viral disease. Symptoms are often not specific hence the importance to confirm the diagnosis during the early stage of the disease. Nevertheless, until recently only specialized laboratories were able to confirm dengue diagnosis. The discovery of the NS1 protein as a marker of infection has allowed the development of point-of-care tests for a rapid diagnosis confirmation. These tests have previously been evaluated by laboratories, but their performances have never been assessed in field conditions. In this study we evaluated the performance of SD Bioline Dengue Duo kit when tests were performed by hospital laboratories staff in a dengue hyper-endemic country. We also assessed the impact of the test results on the clinical management decision. The combination of NS1 test with antibodies detection improved the performance, though discordances on IgM and IgG results were observed between the hospitals and the national reference laboratories. Physicians treated patients according to their clinical diagnosis and did not take negative results into consideration.

## Introduction

The World Health Organization estimates that 50 million dengue infections occur annually and approximately 2.5 billion people live in area at high risk of infection. These areas are located in tropical and sub-tropical regions in South East Asia, Africa, Eastern Mediterranean, Western Pacific, Central and South America. The number of reported cases increased approximately 30 times over the last 50 years [1] and this could be in relation to many factors including population growth, urbanization, failure to control mosquito vectors, etc. [2].

Dengue is a viral disease transmitted by *Aedes* mosquitoes, principally *Ae. aegypti*. Dengue virus (DENV) belongs to the family *Flaviviridae*, genus *Flavivirus*. There are 4 antigenically and genetically distinct serotypes (DENV-1, -2, -3 and -4). In human, the virus can cause a spectrum of illness ranging from asymptomatic infection or self-limiting influenza-like illness (dengue fever or DF) to life-threatening disease associated with vascular leakage, hemorrhage (dengue hemorrhagic fever or DHF), potentially leading to vascular shock (dengue shock syndrome or DSS).

There is currently no specific treatment available for dengue. An early diagnosis is nevertheless very important for efficient clinical management in order to cure or prevent life-threatening complications. In addition, accurate and early diagnosis directs clinical attention to warning signs of an evolution to severe forms and avoids unnecessary use of antibiotics. A range of serological and virological diagnostic methods are available but most of them require specialized laboratory equipment, experienced personnel and are time consuming which is not adapted for a field and point-of-care use. Serological diagnosis by ELISA or rapid diagnostic tests (RDTs) is technically easy to perform and provides fast results but requires most of the time paired sera to definitively confirm the diagnosis [1], [3].

Detection of the NS1 antigen in the blood is a recent and very popular diagnostic method. This viral protein is secreted in the blood and can be detected by ELISA or immunochromatographic tests from the first day of fever and up to 14 days after infection [4]–[7].

The purpose of this study was to evaluate a commercial rapid dengue diagnostic kit, the SD Bioline Dengue Duo device (Standard Diagnostic Inc., Korea), in particular in point-of-care applications, and to evaluate the impact of the results of this combined test on the clinical management decision. The SD Bioline Dengue Duo kit is composed of 2 tests designed to detect DENV NS1 antigen (first test) and anti-DENV IgM/IgG (second test) in serum, plasma or whole blood. The kit evaluation was double. Firstly, the use of the test in the field was for the first time evaluated during a prospective study in 2 Cambodian provincial hospitals. The results obtained in the hospital's laboratories were then compared with those reported with the same samples by a national reference laboratory at Institut Pasteur in Cambodia (IPC). We also investigated how the results of this point-of-care test designed to assist clinical management were perceived and subsequently incorporated into the clinical management decision of physicians from 2 hospitals during a dengue epidemic. Secondly, a more usual retrospective case-control evaluation against reference methods was performed at IPC in order to assess the kit performances in the context of a dengue-endemic South-East Asian country.

## Materials and Methods

### Patients' recruitment and samples collection for the prospective evaluation

Patients were enrolled in the pediatric wards of Kampong Cham and Takeo provincial hospitals during the 2011 dengue epidemic in Cambodia i.e. between June and October 2011. Patients presenting spontaneously to these hospitals or referred by health centers with a history of fever during the previous 7 days and at least one of the following symptoms: rash or severe headache or retro-orbital pain or myalgia or joint pain or bleeding, were examined by physicians who decided whether or not the child should be hospitalized. When the number of beds available was limited, priority was obviously given to the most severe cases. In each hospital, a maximum of 10 hospitalized patients, randomly selected, were enrolled weekly. Patient's information and clinical data were collected by physicians using a specific case report form and blood samples were taken at the time of hospital admission (early/acute specimen) and discharge (convalescent/late specimen). Patients with incomplete test kit results, missing blood samples and incomplete clinical records were excluded.

### Panel of samples used for the retrospective laboratory evaluation

The panel used for the retrospective laboratory evaluation of the kit performances consisted of 157 samples collected in 2011 during the field prospective evaluation and tested negative or positive by the reference methods available at IPC completed with an additional 167 samples selected from IPC's dengue laboratory's biobank (samples collected between 2008 and 2010). Positive samples were selected in order to obtain an evaluation panel as balanced as possible in terms of DENV serotypes, day of collection after onset of fever (DAOF), anti-DENV antibodies titer and immune status (primary/secondary infections). Negative samples were selected from patients presenting with a non-dengue febrile illness and also from pregnant women.

### Ethical aspects

For the field prospective evaluation, a written consent was signed by the children's legal representatives before enrolment. This study was approved by the Cambodian National Ethics Committee. The use of stored samples from IPC's biobank was also approved by the Cambodian National Ethics Committee.

### Dengue diagnosis

The SD Bioline Dengue Duo kits were provided by Standard Diagnostics (Kyonggi-do, Korea) and tests were performed according to the manufacturer's instructions. For the prospective study, only acute blood samples were tested with the kit in hospitals as well as at IPC.

At IPC, laboratory diagnosis was based on RT-PCR, isolation of DENV after inoculation into mosquito cell lines, detection of anti-DENV IgM and measure of an increase of anti-DENV antibodies titer measured by hemagglutination inhibition assay (HIA) between acute and convalescent sera.

RT-PCR was performed after viral RNA extraction from acute serum samples using QIAmp Viral RNA Mini kit (Qiagen, Hilden, Germany). Either a conventional nested RT-PCR according to Lanciotti *et al.* [8] protocol and modified by Reynes *et al.* [9] or a real-time multiplex RT-PCR based on the technique developed by Hue *et al.* [10] was performed.

DENV was isolated on C6/36 cells and the virus serotype identified by immunofluorescence assay using monoclonal antibodies as described previously [11].

An in-house IgM capture Enzyme-Linked Immuno-Sorbent Assay (MAC-ELISA) was used to detect anti-DENV and anti-Japanese Encephalitis virus (JEV) IgM as describe previously [11]. A result was considered positive for dengue when the optical density (OD) was higher than 0.1 for the DENV IgM and when the OD of the anti-DENV ELISA was higher than the OD of the anti-JEV ELISA.

HIA followed the method described by Clark and Casals [12] adapted to 96-well microtiter plate. Primary or secondary acute dengue infection was determined by HI titer according to criteria established by WHO [13]. In brief, the patient was defined as having a primary infection when the convalescent serum had a HI titer ≤2560 associated with a fourfold rise of the titer between the acute and convalescent sera (collected with a time interval of at least 7 days). When the convalescent serum had an HI titer >2560, the patient was defined as having a secondary dengue infection.

All early samples were tested by PCR, viral isolation, IHA and MAC-ELISA whereas late samples were only tested by HIA and MAC-ELISA.

Confirmed and suspected dengue cases were defined according to WHO guidelines [1]. A confirmed case was defined by a RT-PCR and/or a culture positive result and/or an IgM seroconversion in paired sera and/or a fourfold antibodies titer increase measured by HIA in paired sera. A probable dengue infection was defined by an HI antibody titer >2560 in paired sera without a fourfold increase or IgM positive result in the acute serum [1].

At IPC, technicians were blinded for the results of the kit evaluated as well as for the results of gold standard tests. In hospitals, the staff performing rapid diagnostic tests was blinded for the results obtained with these tests as well as for the results of the gold standard assays.

### Hospital case management

Each clinical record contained the complete medical data recorded at the time of admission and the complete follow-up of the patient during the hospitalization (temperature, blood pressure, pulse, diuresis, medical prescriptions, etc.) until discharge. These data were anonymized by the physicians for the purpose of the analysis.

### Statistical analysis

Statistical analysis was performed using STATA version 11.0 (StataCorp, College Station Texas, USA). Significance was assigned at P<0.05 for all parameters and were two-sided unless otherwise indicated. Uncertainty was expressed by 95% confidence intervals (CI95).

For the prospective study, agreement between hospital's laboratories and IPC laboratory's data was measured by agreement percentage and Kappa (κ) coefficient.

For the prospective study, sensitivity and specificity obtained when tests were performed at hospitals were compared with those obtained at IPC with McNemar test. Positive and negative predictive values (PPV and NPV) were compared with Fisher exact test. For the retrospective laboratory study and for the analysis of medical records Fisher exact test was used.

During the retrospective laboratory evaluation, sensitivity was calculated according to infecting serotype, DAOF, immune status and antibodies profiles. Four different antibodies profiles were arbitrarily defined according to HIA and MAC-ELISA results: profile 1, low HI titer (<640) and negative MAC-ELISA; profile 2, low HI titer and positive MAC-ELISA; profile 3, high HI titer (≥640) and negative MAC-ELISA; profile 4, high HI titer and positive MAC-ELISA.

## Results

### Prospective evaluation of the SD Duo kit's performances

#### Characteristics of the study population

A total of 162 patients were enrolled (100 patients in Takeo and 62 in Kampong Cham). The NS1 result of one patient and the IgM/IgG results of 4 others patients were not reported by hospitals. These 5 patients were therefore excluded. At IPC, the reference laboratory tests confirmed 85 dengue cases, 41 children were classified as probable dengue infection, and in 31 cases a dengue infection was excluded. Among the 126 patients with confirmed or probable dengue, 32 were classified as DF, 84 as DHF and 8 as DSS. Clinical, virological and demographical information of the population are summarized in Table 1. All the patients enrolled in this study survived and were discharged without complication or sequelae.

**Table 1:** Characteristics of the patients included in the prospective evaluation.

Variables	Confirmed dengue (n = 85)	Probable dengue (n = 41)	Non-dengue infection (n = 31)
Median age (IQR^a^)	8 (5–11)	10 (7–12)	8 (6–13)
Male (%)	49 (57.6%)	24 (58.4%)	20 (64.5%)
Median day of illness (IQR)	3 (2–4)	3 (3–4)	3 (2–4)
**Dengue diagnostic**			
Virus isolation	21 (24.7%)	0 (0.0%)	-
RT-PCR^b^	72 (84.7%)	0 (0.0%)	-
MAC-ELISA			
*Positive in acute serum*	*48 (56.4%)*	*40 (97.6%)*	*-*
*Seroconversion*	*19 (22.4%)*	*0 (0.0%)*	*-*
*Negative*	*18 (21.2%)*	*1 (2.4%)*	*-*
Hemagglutination-Inhibition assay (titer)			
*Fourfold rise in antibodies on pair sera*	*33(38.8%)*	*0 (0.0%)*	*-*
*No change or less than fourfold rise*	*46 (54.1%)*	*39 (95.1%)*	*-*
*Data not available* c	*6 (7.1%)*	*2 (4.9%)*	*-*
**DENV serotypes**			
DENV-1	53 (62.3%)	-	-
DENV-2	21 (24.7%)	-	-
DENV-3	1 (1.2%)	-	-
DENV-4	0 (0.0%)	-	-
Unknown^d^	10 (11.8%)	-	-
**Clinical manifestation**			
DF	23 (27.1%)	9 (22.0%)	11 (35.5%)
DHF	53 (62.4%)	31 (75.6%)	20 (64.5%)
DSS	8 (9.4%)	0 (0.0%)	0 (0%)
Meningo-encephalitis	1 (1.1%)	1 (2,4%)	
**Diagnosis at hospital discharge**			
Dengue	63 (74.1%)	34 (83%)	23 (74.2%)
Dengue with co-infection	3 (3.6%)	1 (2.4%)	2 (6.4%)
*Bronchiolitis*	*1 (1.2%)*	*0 (0%)*	*0 (0%)*
*Pharyngitis*	*1 (1.2%)*	*0 (0%)*	*0 (0%)*
*Typhoid fever*	*0 (0%)*	*1 (2.4%)*	*2 (6.4%)*
*Nosocomial infection*	*1 (1.2%)*	*0 (0%)*	*0 (0%)*
Non dengue	0 (0%)	1 (2.4%)	2 (6.4%)
*Meningitis*	*-*	*0 (0%)*	*1 (3.2%)*
*Typhoid fever*	*-*	*1 (2.4%)*	*0 (0%)*
*Other viral infection*	*-*	*0 (0%)*	*1 (3.2%)*
Unknown^e^	19 (22.3%)	5 (12.2%)	4 (13%)

a interquartile range.

b 72 samples were tested by the conventional nested RT-PCR and 85 were tested by real-time RT-PCR.

c Absence of second serum.

d Dengue diagnosis based solely on positive serology.

e Medical record missing.

#### Comparison of results between hospital laboratories and Institut Pasteur's laboratory

For the NS1 test, an agreement of 98.1% and a κ coefficient of 0.96 were obtained (Table 2). For the IgM/IgG tests, an agreement of 68.8% and a κ coefficient of 0.55 were observed (Table 3).

**Table 2:** Concordance of the NS1 test between hospitals' laboratories and IPC.

		IPC		
		Negative	Positive	Total
**Hospitals**	**Negative**	98	2	100
	**Positive**	1	56	57
	**Total**	99	58	157

**Table 3:** Concordance of the IgM/IgG tests between hospitals' laboratories and IPC.

		IPC				
		Negative	IgM positive	IgG positive	IgM and IgG positive	Total
**Hospitals**	**Negative**	45	2	9	7	63
	**IgM positive**	0	10	2	7	19
	**IgG positive**	0	0	11	9	20
	**IgM and IgG positive**	3	3	7	42	55
	**Total**	48	15	29	65	157

In the hospitals, the overall sensitivity, specificity, PPV and NPV of the NS1 test were 44.4%, 96.8%, 98.2% and 30% respectively and were 45.2%, 96.8%, 98.3% and 30.3% respectively at IPC. Results were not statistically different (Table 4).

**Table 4:** Performances of the kit against confirmed and probable dengue cases in hospitals and IPC.

Test kit	Place	Sensitivity % [CI95%]	p-value*	Specificity % [CI95%]	p-value*	PPV % [CI95%]	p-value**	NPV % [CI95%]	p-value**
SD Dengue Duo NS1	Hospitals	44.4 (56/126)[35.6–53.6]	1	96.8 (30/31) [83.3–99.9]	1	98.2 (56/57) [90.6–100]	1	30.0 (30/100) [21.2–40]	1
	IPC	45.2 (57/126)[36.4–54.3]		96.8 (30/31) [83.3–99.9]		98.3 (57/58) [90.8–100]		30.3 (30/99) [20.6–39.3]	
SD dengue Duo NS1/IgM/IgG	Hospitals	85.7 (108/126) [78.4–91.3]	0.003	83.9 (26/31) [66.3–94.5]	0.5	95.6 (108/113) [90.0–98.5]	0.49	59.1 (26/44) [43.2–73.7]	0.09
	IPC	94.4 (119/126) [88.9–97.7]		90.0 (27/30) [73.5–97.9]		97.5 (119/122) [93.0–99.5]		77.1 (27/35) [59.9–89.6]	

* Mc Nemar test.

** Fisher exact test.

For the combined test (antibodies and NS1) the overall sensitivity, specificity, PPV and NPV at the hospitals were 85.7%, 83.9%, 95.6% and 59.1% respectively whereas they were 94,4%, 90.0%, 97.5% and 77.1% respectively at IPC. Sensitivity was significantly higher at IPC (p-value = 0.002). Specificity, PPV and NPV were better at IPC but the differences were not statistically significant (Table 4).

The combination of all the tests significantly improved the sensitivity and NPV of the kit at hospitals (p-value sensitivity <0.001; p-value NPV = 0.001) as well as at IPC (p-values <0.001) with a non-significant decrease of the specificity (p-value at hospitals = 0.12, p-value at IPC = 0.5) and of the PPV (p-value at hospitals = 0.67, p-value at IPC = 1).

### Retrospective laboratory evaluation of the SD Duo kit's performances

#### Samples description

A total of 166 positive samples and 120 negative samples were included in the retrospective laboratory evaluation of the kit. Eighty five and 81 positive samples as well as 31 and 89 negative samples were obtained from the 2011's prospective study and from the IPC's biobank, respectively. The 41 patients defined as suspect dengue infection by the gold standard methods during the prospective study were also included in the retrospective study. Positive samples included 86 sera with a low HI titer (<640) and 80 sera with a high HI titer (≥640) (51.8% and 48.2%, respectively). The distinction between low and high HI titer groups was established during a preliminary comparative study between HI titers and SD Duo kit IgG results. In the high HI titer group, the correlation between both tests was over 70% which was considered as acceptable (data not shown). Of note, HIA does not only detect IgG but also other immunoglobulin isotypes and as such some low HI titers measured during the early phase of the infection may not contain or only very low quantities of anti-DENV IgGs. DENV serotype was identified in 87.3% of the positive samples: there were respectively 57, 35, 26 and 27 (34.3%, 21.1%, 15.7% and 16.3%) DENV-1, -2, -3 and -4 cases. Forty seven (28.3%) samples were collected 2 days after onset of fever or earlier, 67 (40.4%) between day 3 and day 4, 41 (24.7%) between day 5 and 6, and 11 (6.6%) after the 7^th^ day of illness (Table S1). A total of 19 and 83 patients (11.4% and 50%) were classified as having primary and secondary infections, respectively.

Negative samples included sera obtained from patients infected by a *Plasmodium vivax or P. falciparum* (22/120, 18.4%), *Orientia tsutsugamushi* (13/120, 10.8%), Japanese encephalitis virus (16/120, 13.3%), Chikungunya virus (14/120, 11.7%), Hepatitis C virus (10/120, 8.3%), from patients presenting with meningo-encephalitis (6/120, 5%) or non-specific febrile illness of unknown etiology (30/120, 25%) and from pregnant women (9/120, 7.5%).

#### NS1 test

The overall sensitivity and specificity of the NS1 test against the reference methods was 58.4% (97/166, CI95 = [40.5–66.0]) and 98.3% (118/120, CI95 = [94.1–99.8]), respectively (Table 5).

**Table 5:** Sensitivity of individual and combination of tests according to serotype and immune status.

	Sensitivity % [CI95%]					
	NS1 test	p-value	IgM/IgG test	p-value	NS1 and IgM/IgG tests	p-value
**Global**	58.4 (97/166) [40.5–66.0]		68.1 (113/166) [60.4–75.1]		94.6 (157/166) [90.0–97.5]	
**Serotype**						
DENV-1	61.4 (35/57) [47.6–74.0]	0.129	70.2 (40/57) [56.6–81.6]	0.238	91.2 (52/57) [80.7–97.1]	0.868
DENV-2	48.6 (17/35) [31.4–66.0]		68.6 (24/35) [50.7–83.1]		94.3 (33/35) [80.8–99.3]	
DENV-3	65.4 (17/26) [44.3–82.8]		65.4 (17/26) [44.3–82.8]		96.1 (25/26) [80.4–99.9]	
DENV-4	77.8 (21/27) [57.7–91.4]		48.2 (13/27) [25.5–64.7]		100 (27/27) [87.2–100]^a^	
**Immune status**						
Primary infection	89.5 (17/19) [66.9–98.7]	<0.001	42.1 (8/19) [20.3–66.5]	0.003	100 (19/19) [82.4–100]^a^	1
Secondary infection	43.4 (36/83) [32.5–54.7]		79.5 (66/83) [69.2–87.6]		97.6 (81/83) [91.6–99.7]	

a one-side, 97,5% confidence interval.

Sensitivity was significantly better for DENV-4 than DENV-2 (77.8% vs 48.6%, p-value = 0.01). No differences were observed with the other serotypes (Table 5). Sensitivity was also significantly better in primary than in secondary infections (89.5% vs 43.4%, p-value<0.001) (Table 5). When considering the various antibodies profiles, the analysis demonstrated that a high HI titer (profiles 3 and 4) was associated with a significant decrease in NS1 test's sensitivity (profile 1 vs 3, p-value = 0.005; profile 1 vs 4, p-value<0.001; profile 2 vs 3, p-value = 0.014; profile 2 vs 4, p-value<0.001). The presence of detectable IgM (profiles 2 and 4) did not seem to affect the sensitivity (profile 1 vs 2, p-value = 1; profile 3 vs 4 p-value = 0.185) (Table 6).

**Table 6:** Sensitivity of SD Bioline Dengue Duo NS1 test according to antibodies profile.

	Profile 1 (Low HI titer/IgM negative)	Profile 2 (Low HI titer/IgM positive)	Profile 3 (High HI titer/IgM negative)	Profile 4 (High HI titer/IgM positive)
**Sensitivity % [CI95%]**	83.7 (41/49) [70.3–92.7]	83.3 (25/30) [65.3–94.4]	50.0 (9/18) [26.0–74.0]	32.8 (19/58) [21.0–46.3]
**p-value**	1*		0.185**	

* P-value refers to the comparison between profile 1 and 2.

** P-value refers to the comparison between profile 3 and 4.

**Figure 1:**
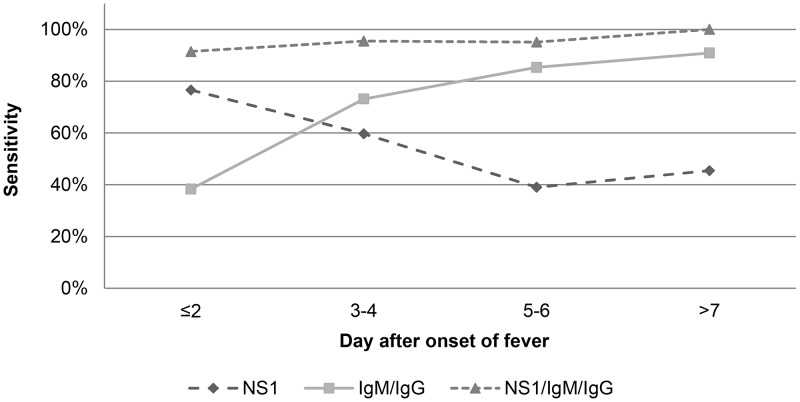
Sensitivity of SD Bioline Dengue Duo kit. Evaluation of the sensitivity of NS1 test, IgM/IgG test and combination of the two tests depending on day of sampling after onset of fever.

The test's sensitivity diminished significantly also when the interval of time between onset of fever and sample collection increased (overall p-value = 0.003). This value varied from 76.6% (36/47, CI95 = [62.0–87.7]) for samples collected before the 2^nd^ day of illness to 45.5% (5/11, CI95 = [16.7–76.6]) for those collected after the 7^th^ day of illness (Figure 1).

Out of the 41 patients with only suspected dengue infection as defined by the reference diagnostic methods used, 12 (29.2%) tested positive by NS1 kit. Out of the 120 negative samples, two weak signals were recorded, one from a patient presenting with a febrile illness of unknown etiology and one from a patients with a JEV infection.

#### IgM/IgG tests

The overall sensitivity and specificity of the IgM/IgG tests were 68.1% (113/166, CI95 = [60.4–75.1]and 95% (114/120, CI95 = [89.4–98.1]), respectively (Table 5).

The sensitivity was better with DENV-1 than DENV-4 but this difference was at the limit of significance (70.2% vs 48.2%, p-value = 0.051). No differences were observed with the other serotypes (Table 5). The sensitivity improved significantly when the interval of time between onset of fever and sample collection increased (overall p-value<0.001), from 40.4% (18/47, CI95 = [26.4–55.7]) for samples collected before the 2^nd^ day of illness to 90.9% (10/11 CI95 = [58.7–99.8]) for those collected after the 7^th^ day of illness (Figure 1).

The sensitivity was also significantly better in patients with secondary than primary infections (79.5% vs 42.1%, p-value = 0.003) (Table 5).

All the 41 patients with a suspicion of DENV infection tested positive either by IgM only (6/41, 14.6%), by IgG only (11/41, 26.8%) or by both IgM and IgG (26/41, 63.4%). In 3 non-dengue febrile cases and 3 anti-JEV IgM positive cases the kit gave non concordant results compared to our reference methods. In 2 non-dengue febrile cases both IgM and IgG were positives. In 1 JEV case the IgG test was positive while in 1 non-dengue febrile and 1 JEV cases, IgG test was weakly positive. In the last JEV case, the IgM test was weakly positive.

#### NS1 and IgM/IgG tests combination

The overall sensitivity of the NS1/IgM/IgG combination tests was 94.6% (157/166 CI95 = [90.0–97.5]) and the specificity was 94.2% (113/120, CI95 = [88.4–97.6]) (Table 5).

The sensitivity did not vary significantly between the serotypes when compared all together (p-value = 0.868), or 2 by 2, but also not according to the immune status (p-value = 1) or the time interval between DAOF and sample collection (p-value = 0.8) (Table 5, Figure 1).

### Impact of the RDTs results on clinical case management in Cambodia

The medical records of 129 patients (82.2% of all patients enrolled) were provided by the two hospitals and subsequently analyzed. All the 66 patients who tested positive for acute dengue infection using the IPC gold standard test were also clinically diagnosed by the physicians as dengue cases, with or without co-infection (63 and 3 patients, respectively). One patient with a laboratory-suspected DENV infection as well as two children who tested negative were clinically diagnosed as non-dengue febrile illness (Table 1).

All patients received a treatment based on WHO 2009 recommendations, i.e., intravenous fluid therapy with 0.9% saline, Ringer's lactate or Ringer's acetate with or without dextrose, paracetamol if fever and oral rehydration solution or other fluids containing electrolytes and sugar when possible. Patients in circulatory shock received dextran, O_2_ and blood transfusion when necessary. Twenty-nine patients (27.7%) also received antibiotics. The prescription of antibiotics was justified by the phisicians in the medical records of 11 patients because the following diagnoses: 4 dysenteric syndromes with suspicion of typhoid fever, 3 meningitis or meningo-encephalitis, 1 suspicion of nosocomial infection, 2 pharyngitis and 1 bronchiolitis. Among the 90 patients with a positive NS1 and/or IgM and/or IgG test, 17.8% (16/90) were treated with antibiotics. Out of 39 patients who tested negative by the RDT, 13 (33.3%) also received antibiotics. The comparison of antibiotic prescription between both groups was at the limit of significance (p-value = 0.067). There was no difference in the duration of antibiotic therapy between patients with a positive test and those with a negative test (p-value = 0.216).

Among the 16 positive patients who received antibiotics, only 7 (43.7%) had their antibiotic therapy stopped once the point-of-care kit tested positive for dengue. Among the 13 patients with a negative result who received antibiotics, 8 (61.5%) had their antibiotic therapy stopped once the test was performed. The decision to maintain or discontinue the antibiotic therapy was not affected by the result of the RDT (p-value = 0.338).

## Discussion

Early management of patients with dengue infection is essential to ensure a favorable evolution of the disease and prevent the occurrence of severe forms. Until recently an early confirmed diagnosis was only achievable in specialized laboratories. The discovery of the NS1 protein as an early marker for DENV infection, especially in RDT format, now allows dengue diagnosis during the early phase of the disease, even in laboratories with limited equipments and human resources. Evaluations are required to ensure that these tests are suitable for diagnosis and clinical management or epidemiological surveillance and outbreak investigations. Different methodologies can be used: laboratory-based evaluations (or retrospective evaluations) and field evaluations (or clinical-based/prospective evaluations) [14]. Retrospective evaluations are easy to perform but tend to overestimate tests accuracy. Prospective evaluations allow determination of PPV and NPV with tests performed on patients in the real clinical settings. However, accuracy of diagnostic tests estimated by prospective evaluations could be biased due to imperfect gold standard in the prospective clinical setting. In our study we combined both prospective and retrospective evaluations. The retrospective part was added in order to better understand the results obtained in the field during the prospective study.

Since the two test kits of the SD Bioline Dengue Duo combo test do not give exactly the same information, the NS1 assay was initially assessed alone in the prospective as well as in the retrospective study. If a positive NS1 test can confirm a dengue diagnosis, this is not the case for IgM and IgG tests as the antibodies remain detectable for months and thus a positive result obtained on a single blood specimen is only suggestive of a dengue infection. Indeed, to confirm an acute dengue infection by serology, an IgM seroconversion or a four-fold increase of IgG antibody titers in paired sera must be demonstrated (which cannot be done with the RDT kit as result is only qualitative) [1]. By evaluating separately, but in parallel, the NS1 test and the serological kit, we estimated the ability of the test to both suggest and confirm a dengue infection.

During the prospective study, the sensitivity of the SD Bioline Dengue Duo NS1 when performed at the hospitals was only 44.5% to confirm dengue infections in children hospitalized for dengue-like illness during the epidemic season. The tests were carried out in laboratories equipped for routine medical biology. Out of the 127 patients included in the prospective evaluation, 70 (54.7%) had an HI titer ≥640 which could probably explains such a poor sensitivity. The retrospective study helps to understand why the sensitivity was limited. It suggested that the presence of high level of anti-DENV HI antibodies in the sample was a major factor for sensitivity decrease. Indeed, while a sensitivity >80% was obtained with samples containing no or low HI antibodies titer (<640), the sensitivity dropped to 37% when the HI titer was ≥640. Almost 86% of the samples with a high HI titer issued from patients with a secondary infection. Since HI titer reflects mainly IgG response, the poor sensitivity observed during secondary infections is probably directly linked to the high IgG titer. Similar observations were already made by other authors. In Vietnam, the same NS1 test demonstrated a sensitivity of 24.6% for samples positive for IgG by GAC-ELISA and a sensitivity of 77.3% in sera negative for IgG [15]. In Colombia, Osario *et al.* reported an even lower sensitivity (IgG negative: 65.6%, IgG positive: 15.6%) [16]. Of note, the methods used for IgG detection in these evaluations were all different and rather than giving the real performance of the kit, the data indicate a global trend to a lower sensitivity when IgG titers increase.

As others [16], [17], we observed that the sensitivity of this test decreased when the window of time between onset of fever and sampling increased. This was expected since the IgG titer also increased with the time. Finally, a higher IgG titer also characterizes the secondary dengue infections and the better sensitivity of the NS1 in primary infections was also already reported [15]–[17].

The performances of the NS1 test reported here as well as by other retrospectives studies are close to those observed with other commercial NS1 RDTs [15], [18]. A major value of the kit marketed by SD is the combination of the NS1 test with an anti-DENV antibodies detection kit. Indeed, the serological results improved the sensitivity by compensating for the loss of sensitivity usually observed with the NS1 test when used alone in the presence of specific anti-DENV antibodies. During the prospective evaluation, we demonstrated that the addition of IgM and IgG results to the NS1 data was only associated with a slight non-significant decrease of the specificity. However, this result should be interpreted with caution as the number of negative patients included was relatively small. In addition, the relatively low overall performance of the IgM/IgG test could well be partially due to imperfect gold standard tests. In the retrospective study, we did not observed any cross-reactivity with Chikungunya virus, *Orientia tsutsugamushi* or *Plasmodium sp..* However when evaluating the SD Bioline Dengue Duo kit, Blacksell et al. [18] reported 12.2% (10/82) of cross-reactivity with Chikungunya virus, 12.5% (1/8) with *Orientia tsutsugamuhi* and 100% (1/1) with *Plasmodium sp.* When evaluating only the IgM part of the kit, Hunsperger et al. [19] reported around 35% of IgM cross-reactivity with malaria as well as some false positive results with leptospirosis, tuberculosis and West-Nile infections.

During the prospective evaluation, the PPV value of the NS1 test was 98.2%, suggesting that the probability to correctly confirm a dengue infection was very high when the test was positive. When the test was used in combination, the PPV decreased only very slightly (NS1/IgM: 96.9%; NS1/IgM/IgG: 95.6%). Consequently, the NPV observed when the tests were performed in the hospitals was only 29% for the NS1 test alone and 56.8% for the combination test. In other words, the probability of truly exclude a dengue infection when the tests were negatives was low. These PPV and NPV results should be regarded with caution as they depend on the dengue disease prevalence that can be extremely different in other contexts and epidemiological situations. In this prospective study, the prevalence of dengue infection was very high (80.3%, 126/157) because the evaluation was performed during the peak epidemic season and only involved dengue suspect patients. Observing high prevalence of dengue infections in suspect patients hospitalized is common in Cambodia (87.8% of average between 2000 and 2008) and in neighboring countries like Vietnam (86.2% during a DENV-4 epidemic in 2002) [20], [21].

On the samples collected during the prospective study, the comparison of the results of the tests performed by technicians in hospital laboratories or by health workers who did not receive any specific training for the use of the kits with the results reported by the staff of the national reference laboratory at IPC demonstrated a moderate agreement with the serological tests and an excellent agreement with the NS1 test. Indeed, 49 discordant results between the hospitals and IPC were observed with the IgM/IgG test out of which 34 (69.3%) were positive at IPC but negative at the hospitals while 13 (26.5%) were negative at IPC but positive at the hospitals. These discrepancies could be explained if the reading was made before the recommended 15 minutes (leading to false negative results) or after the correct time (leading to apparition of unspecific bands) or because of problems with the interpretation of weak signals (faint bands). To evaluate if the issue was the interpretation of the faint bands, these data were removed from the analysis and a better agreement percentage and Kappa coefficient were obtained (82.0% vs 68.8% and 0.73 vs 0.55). A problem of reproducibility could also have accounted for some of the discrepancies observed. Nevertheless, in the case of bad reproducibility an equal number of discrepancies should have been observed in each laboratory which was not the case in our study. Moreover all tests were from the same manufacturing lot. During a malaria RDTs evaluation, misinterpretation of weak signal in the field had already been reported [22]. It was also reported that health workers in the field tend to read the results before the time recommended by the manufacturer [22], [23]. Despite its relative ease to use, the performances of the IgM/IgG RDT are obviously partially person-dependent, hence the importance of providing specific training or at least very clear pamphlets which could guide the health worker in its interpretations and expose the risks of false results when the recommendations are not strictly followed. On the contrary a very good agreement was observed with the NS1 test since the bands in this immunochromatographic device almost always appear very clearly. As the RDTs have a significant cost, promoting the use of these kit does only make sense if the health workers can perform the tests in good conditions, which seems to be sometimes challenging in intensive care units and pediatric wards that are often unable to cope during peak epidemics. Knowing these constraints and limitations, the manufacturer should be encouraged to correct, if possible, the reading issues of the serological test. The outcomes of the patients who were wrongly tested negative by the kit was a matter of concern as RDTs are designed for rapid diagnostic and to assist physicians in their decisions. Dengue is a life-threatening disease that requires specific clinical care. The analysis of the medical records demonstrated that physicians ignored the negative results and followed their clinical instinct as all patients who tested negative by RDT received an intravenous fluid therapy which is recommended in patients with warning signs [1] but which is also often administrated in mild cases to prevent complications. Similar observations were also made in the context of malaria RDTs use. Between 54% and 85% of the patients with negative malaria RDT results were treated with anti-malaria drugs in Nigeria, Tanzania, Burkina Faso, Philippines and Laos [22]–[25]. There are probably several reasons that could explain that physicians did not consider the negative results obtained with the RDT: the habit to rely mostly on clinical intuition explained by a frequent limited access to laboratory tests, some mistrust against a new test, the difficulties to understand the kinetic of the immune response during dengue infection and the significance of NS1, IgM and IgG test results, a high confidence in clinical diagnosis when children present to pediatric wards with dengue-like symptoms during the epidemic season (especially since the national virological surveillance confirms usually more than 80% of the dengue clinical diagnosis) [20], the fear that a misdiagnosed dengue infection evolves towards a DHF or a DSS while these complications are pretty easy to prevent with simple clinical management, etc. The SD Bioline dengue Duo test could have a better utility in smaller medical care structures, like health care centers and dispensary where the proportion of dengue among all febrile diseases is lower (e.g., 12% of all the febrile episodes in Kampong Cham province, 2006–2008) [11] and where routine hematology (e.g., hematocrit, platelet count) that could help to orientate the diagnosis are not often available.

One of the advantages to perform a rapid confirmatory diagnostic of dengue in the context of febrile illness is to avoid the unnecessary use of antibiotics. In the context of Cambodia, it seems the RDT results did not have a significant impact on the decision to start or discontinue an antibiotic therapy.

In an endemic country, especially in the context of an epidemic, it seems that the sensitivity of the NS1 RDT alone is too low and that only positive results should be taken into consideration. Nevertheless, the performances of the combined kits are good and these kits appear to be a useful tool for the clinicians as they can quickly confirm the diagnosis of dengue and therefore contribute to the an optimal clinical management of the cases and avoid an unnecessary use of antibiotics or other drugs which is important in the context of a developing country with limited resources.

In conclusion, we observed that for a patient presenting with dengue-like symptoms in a dengue-endemic/epidemic region, a NS1 positive result obtained with the SD Bioline Dengue Duo kit confirms a dengue diagnosis, an IgM and/or IgG positive result highly suggests dengue infection but a negative result doesn't rule out a dengue infection. We have also demonstrated that the performances of the test in the field were lower than the ones obtained in the more experienced hands of technicians working in a national reference laboratory. This suggest that even for a point of care test theoretically designed to be used by untrained staff, there is still a significant improvement of the performance of the test to expect if a proper training and a quality assurance program can be implemented. With the time, the trust of the physician will probably increase if the accuracy of the test improves. In general, manufacturers should always bear in mind that the ultimate goal of the RDTs is essentially to be used as a point-of-care test or in support of epidemiological investigation and as such should be easy to use, stable at room temperature but also not posing reading difficulties unless they can provide proper training and organize quality programs. More prospective field evaluations are still necessary now to better assess the interest to use such point-of-care tests in the real conditions that justified their development and to address some of the questions and concerns raised by this study.

## Supporting Information

Table S1 **Composition of the panel of samples used for retrospective evaluation.** Description of the panel of positive samples used for the laboratory retrospective evaluation of SD Bioline Dengue Duo Kit.
(DOCX)
