# Supplementary material for: Field Evaluation and Impact on Clinical Management of a Rapid Diagnostic Kit That Detects Dengue NS1, IgM and IgG
Source: PLoS Negl Trop Dis. 2012 Dec 27;6(12):e1993. doi: 10.1371/journal.pntd.0001993 (PMC3531494; doi:10.1371/journal.pntd.0001993)
Supplement: Table S1 — Composition of the panel of samples used for retrospective evaluation. Description of the panel of positive samples used for the laboratory retrospective evaluation of SD Bioline Dengue Duo Kit. (DOCX) [file pntd.0001993.s001.docx]

|  |  | **Day after onset of fever** | | | |  |  |
| --- | --- | --- | --- | --- | --- | --- | --- |
| **HI titer** | **Serotype** | **≤ 2** | **3 – 4** | **5 – 6** | **≥ 7** | **Total** | |
| <640 (n=79) | DENV-1 | 14 | 10 | 9 | 1 | 34 | |
|  | DENV-2 | 6 | 7 | 3 | 0 | 16 | |
|  | DENV-3 | 4 | 6 | 4 | 0 | 14 | |
|  | DENV-4 | 3 | 8 | 1 | 0 | 12 | |
|  | Unknown | 0 | 0 | 0 | 3 | 3 | |
| ≥640 (n=87) | DENV-1 | 5 | 9 | 8 | 1 | 23 | |
|  | DENV-2 | 5 | 9 | 4 | 1 | 19 | |
|  | DENV-3 | 2 | 8 | 2 | 0 | 12 | |
|  | DENV-4 | 5 | 4 | 6 | 0 | 15 | |
|  | Unknown | 3 | 6 | 4 | 5 | 18 | |
|  | Total | 47 | 67 | 41 | 11 | 166 | |

**Table S1. Description of the panel of positive samples used for the laboratory evaluation of SD Bioline Dengue Duo test**
